# Flexible versus standard intramedullary rod in posterior stabilized primary total knee arthroplasty: protocol for a randomized controlled trial

**DOI:** 10.1186/s13018-020-01989-9

**Published:** 2020-10-14

**Authors:** M. R. Bénard, R. F. M. van Doremalen, A. B. Wymenga, P. J. C. Heesterbeek

**Affiliations:** 1 Sint Maartenskliniek Research, Sint Maartenskliniek, Nijmegen, The Netherlands; 2 Robotics and Mechatronics, University of Twente, Enschede, The Netherlands; 3 Sint Maartenskliniek Orthopaedics, Sint Maartenskliniek, Nijmegen, The Netherlands

**Keywords:** Total knee arthroplasty, Intramedullary rod, Sagittal fit, Functional outcome, Femoral bowing

## Abstract

**Background:**

In total knee arthroplasty (TKA) a flexible intramedullary rod can be used to account for sagittal bowing of the distal femur. Although patients report better post-operative functional outcome when the flexible rod was used, it is unknown how the use of the flexible rod affects the placement of the femoral TKA component, and how this relates to activities of daily living. It is expected that the use of the flexible rod will result in a more flexed femoral component, a larger patellar tendon moment arm, and consequently in better functional outcome. The goal of this study is to compare the flexible rod to the standard intramedullary rod in primary TKA in terms of fit of the TKA, functional outcome, and sizing of the femoral component.

**Methods:**

A single-blind randomized controlled trial with two groups (flexible vs standard rod), with patients blinded for group allocation, and 2 years post-operative follow-up. The fit of the TKA is quantified by two parameters: (1) the flexion angle of the TKA in the sagittal plane and (2) the sagittal profile of the distal femur compared between the pre-operative bone and the TKA. Both parameters are calculated in 3D volume images obtained using fluoroscopy. Functional outcome will be measured using (1) the timed Get-up and Go test (2), the stair climbing test (3), knee power output, and (4) patient and clinician reported outcomes. Different parameters will be measured during the TKA procedure to account for the invisibility of cartilage in the 3D volumes and to study if the amount of bone removed during the procedure is affected by group allocation.

**Discussion:**

The sagittal fit of TKA is not a standardized outcome measure. We discuss our choice of parameters to define the sagittal fit (i.e., flexion angle and sagittal profile), our choice for the parameters we measure during the TKA procedure to account for the lack of cartilage thickness in fluoroscopy, and our choice for the parameters to study if the amount of bone removed during the procedure is affected by group allocation. Lastly, we discuss the merits of this planned trial.

**Trial registration:**

Netherlands Trial Register, 4888, registered 30 March 2015. https://www.trialregister.nl/trial/4888

## Background

Total knee arthroplasty (TKA) is a cost-effective surgical procedure for degenerative knee osteoarthritis. Although TKA has good long-term results, there are still some patients with poor long-term results [13]. Also, the good results are not always related to patient satisfaction and functional outcome [3, 7]. This may be caused by the fact that the kinematics of the TKA do not properly mimic the kinematics of the natural knee. Manufacturers of TKAs have addressed this by integrating aspects of natural knee kinematics into their TKA designs and instruments [9].

One aspect influencing natural knee kinematics is sagittal placement of the femoral component [18]. The instruments used for alignment of the femoral component are classically used intramedullary, and implanting the femoral component can be performed either according to the longitudinal axis of the femur in the sagittal plane or according to the anatomy of the distal femur, taking into account sagittal bowing of the femur. The amount of sagittal bowing can differ between individuals [30], and therefore to account for sagittal bowing during placement of the femoral component, a flexible intramedullary rod (flex rod) can be used instead of the standard intramedullary rod [17].

Up till now, the literature has not been conclusive which of the rods should be used, and acknowledges the dilemma of implanting the femoral component either according to the anatomy of the distal femur, or according to the longitudinal axis of the femur in the sagittal plane, thus ignoring sagittal bowing. The standard rod is considered a safe option. However, it is hypothesized that with the flex rod, less bone needs to be removed because the femoral component better fits to the natural anatomy of the distal femur in the sagittal plane. This would lead to more natural femorotibial kinematics through increasing the patellar tendon moment arm, and thus the possibility for larger knee power output without influencing muscle strength. This effect has been found in a modeling study [24] and could potentially lead to better functional outcome in vivo. Indeed, patient-reported functional outcome is higher up till 2 years in patients operated using the flex rod when compared the standard rod [17]. It is expected that due to less oversizing in the TKA procedure using the flex rod activities of daily living (ADL) such as stairclimbing, and rising from a chair can be performed more optimally [6]. Marra et al. have also shown in their modeling study that less oversizing resulted in better patellofemoral kinematics during rising from a chair [24]. It is also expected that with the use of the flex rod, the proper fit of the TKA will result in an overall smaller size chosen by the surgeon, as has been found in a retrospectively analyzed cohort and a modeling study [8, 17].

## Methods/design

### Aim

The primary aim of this study is to compare the flex rod with the standard rod in terms of post-operative sagittal fit of the distal femur. In addition, sizing and post-operative functional outcome will be evaluated.

### Design and setting

A single-blind randomized controlled trial with two groups (flex rod vs standard rod), with the participants blinded for group allocation. Participants will be measured on five occasions in our outpatient clinic (pre-operative, per-operative/post-operative, 3 months, 1 year, and 2 years post-operative). The study ends after 2- year follow-up. The trial has been set up according to CONSORT guidelines [29], and ethical approval for this study was obtained from *Medisch-ethische toetsingscommissie Slotervaartziekenhuis en Reade* (approval ID P1453).

### Participants

Subjects will be selected from the waiting list for patients scheduled for elective TKA in our hospital. Inclusion and exclusion criteria are shown in Table 1. Eligible subjects will be contacted, after their surgeon’s consent, by phone by a research nurse explaining the study. When they are interested in participating, the patient information sheet will be send. Eligible patients are contacted again after a minimum of 1 week for verbal consent. Written informed consent is obtained at the first study contact in our outpatient clinic.

**Table 1:** The inclusion and exclusion criteria of the subjects

Type	Description
Inclusion criteria	• Patient with non-inflammatory knee osteoarthritis which is radiologically confirmed and which requires total knee replacement. • Age between 40 and 75 years, inclusive. • Patient plans to be available for follow-up until 2 years post-operative. • Patient is in stable health and is free of or treated for cardiac, pulmonary, hematological, or other conditions that would pose excessive operative risk. • Patient has < 10° fixed (non-correctable) varus or valgus deformity of the knee.
Exclusion criteria	• Patient has a BMI > 35. • Patient’s expected physical activity after surgery is 2 or less on the UCLA Activity Scale. • Patient has had previous hip or knee replacement surgery in the last 6 months or is planned to have a (second) hip or knee replacement in the next 6-12 months (because of the effect on function). • Patient has had a previous hip replacement on the affected side (this may cause for a restriction for the rod placement during surgery). • Patient has had major, non-arthroscopic surgery to the study knee, including HTO. • Patient has an active, local infection or systemic infection. • Patient has physical, emotional, or neurological conditions that would compromise compliance with post-operative rehabilitation and follow-up. • Bone stock compromised by disease, infection, or prior implantation which cannot provide adequate support and/or fixation to the prosthesis. • Severe instability of the knee joint secondary to the absence of collateral ligament integrity and function. • Severe instability of the knee joint due to loss of cartilage reported as “substance loss.” • Patient has knee flexion < 90°. • Patient has fixed flexion deformity >10° (passive extension lag). • Patient has >30° extension deficit (active restraint to extension). • Patient does not have a proper functioning patella tendon on the affected side measured as inability of active extension of the knee. • Patient has quadriceps weakness on the affected side; score on MRC scale < 4. • Patient has rheumatoid arthritis, any auto-immune disorder, or immunosuppressive disorder.

### Description of materials

All participants will receive the posterior stabilized model Triathlon (Stryker, Mahwah, NJ, USA). This model is used to standardize the posterior placement of the femoral component (in contrast to a cruciate-retaining model), which is expected to yield differences in the anterior part of the distal femur and therefore differences in patellofemoral function between the groups.

### TKA procedure

**Fig. 1:**
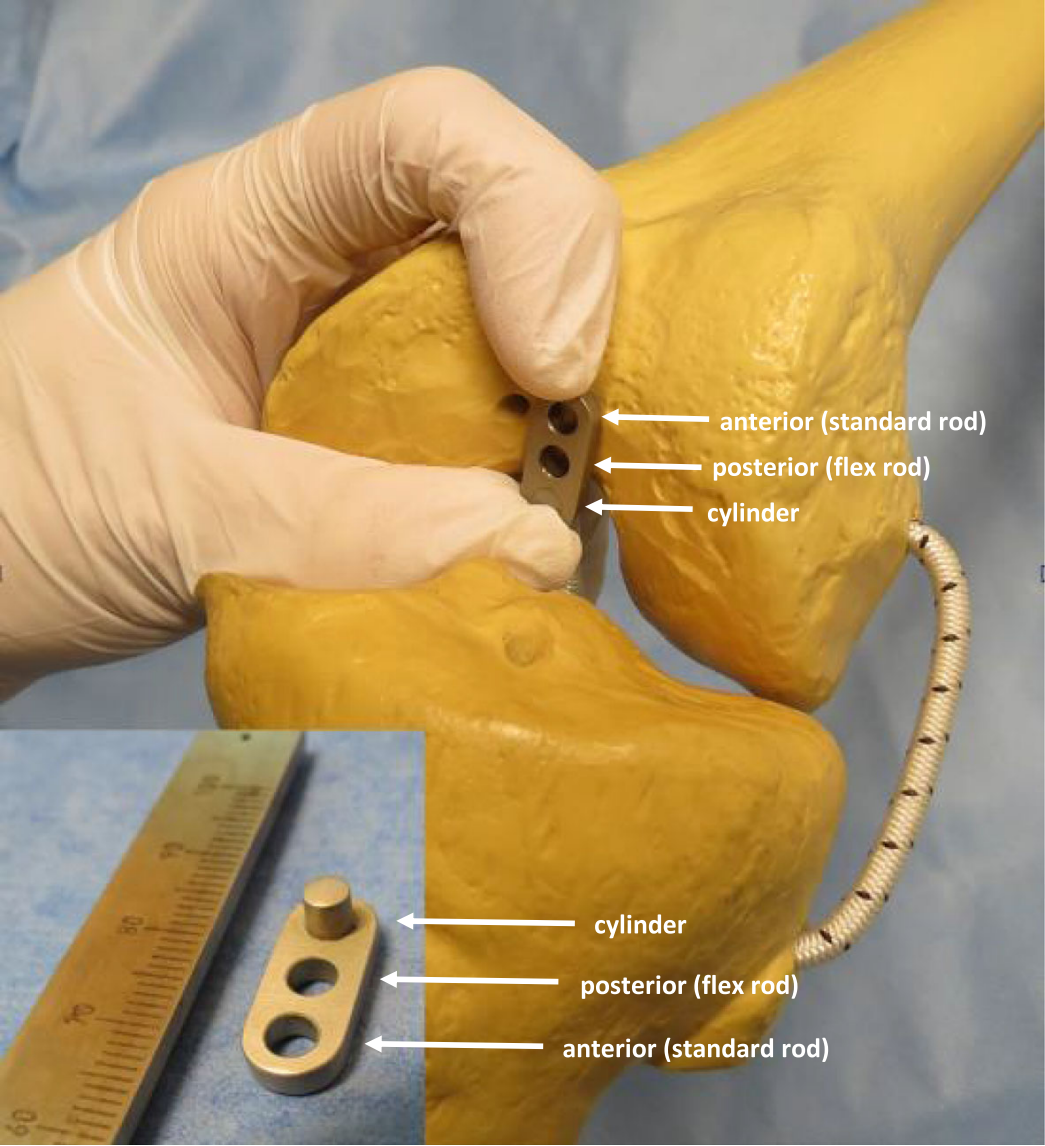
The custom-made aiming device used to standardize the distal entry points of the two rods. The device has a small cylinder which is placed in the intercondylar notch, and two holes, all interspaced at 5 mm. The anterior and posterior holes are used to mark the entry point for the standard rod and flex rod, respectively

The surgical technique is a standardized bone-referenced technique. A tibial bone cut is made based on the standard extramedullary jig from the system aiming at resection of 9 mm bone from the unworn lateral side, or 3 mm below a worn medial compartment. The amount of valgus of the distal cut femur jig is set on the femur angle measured on hip-knee-ankle radiographs of the individual patient. A special custom-made aiming device with an offset of 5 mm (for the flex rod) and 10 mm (for the standard rod) referencing from the intercondylar notch is used to standardize the entry points of the two rods (Fig. 1). Sizing of the femur component is done with posterior referencing. Standard 3° of femoral external rotation is used in these series. Necessary soft tissue releases are done for gap balancing. A standard patella resurfacing is done, and the total implant is placed cemented.

### Investigational product

**Fig. 2:**
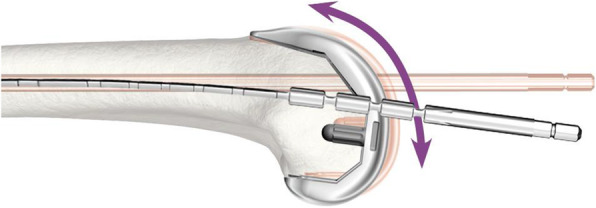
The difference between the standard rod (shaded in background) and the flex rod (clear in foreground) shown in the sagittal view of the distal femur

For this study, the difference between the two groups is the use of the flex rod or the standard rod during TKA. The rods are used in TKA to position the femoral component in the correct sagittal orientation (Fig. 2). The difference between the two rods is that the flex rod allows for bowing in the sagittal plane, thus following the natural anatomy of the femur. There are no extra risks involved with use of the flex rod. Both rods are part of the operating instruments used for placement of the TKA (Stryker, Mahwah, NJ, USA).

### Study parameters

**Fig. 3:**
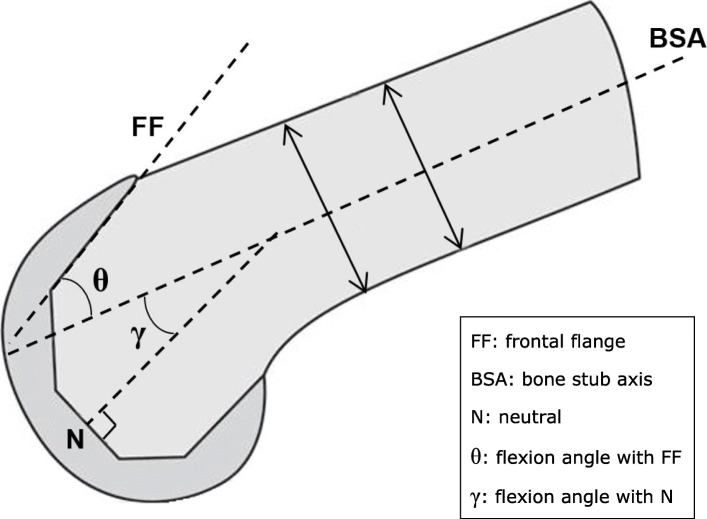
The flexion angle (*θ*) of the prosthesis. This angle is measured in the sagittal plane between the longitudinal bone stub axis (BSA) and the frontal flange (FF). The double arrows are used for determining the orientation of the BSA. The angle *γ* between the neutral line of the femoral component (*N*) and the BSA is calculated by *γ* = *θ* − 7°

**Fig. 4:**
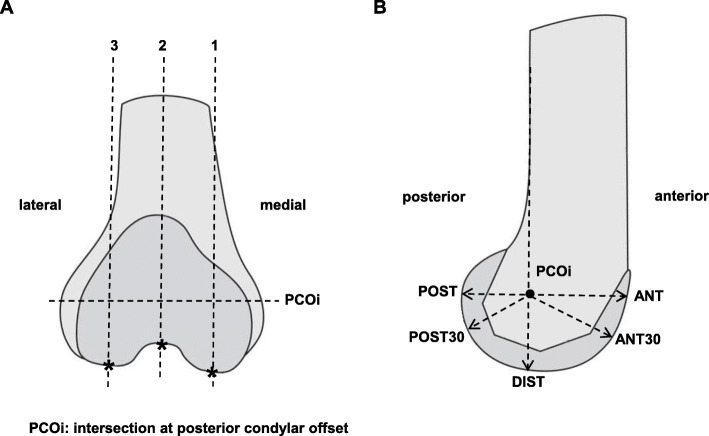
**a** Sagittal contour of the distal femur. The sagittal profile lines are oriented with between the intersection of the posterior condylar offset (PCOi) and the outer border of the native bone or femoral component (shown here). For the profile, five lines are plotted: straight anterior (ANT), anterior at 30° (ANT30), straight distal (DIST), posterior at 30° (POST30), and straight posterior (POST). **b** The orientation of the 3 sagittal slices of the femur where the sagittal profile is calculated: (1) the medial slice, (2) the trochlea slice, and (3) the lateral slice. All slices are oriented perpendicular to the line through the PCOi (see **a**)

The fit of the femoral component is quantified in two parameters: the flexion angle of the TKA in the sagittal plane (Fig. 3), and the sagittal profile at three slices: medial, lateral, and at level of the trochlea (Fig. 4). Both parameters will be measured in a 3D volume image of the knee, reconstructed from images obtained with fluoroscopic assessments using the MultiDiagnost Eleva (MDE, Philips, Amsterdam, The Netherlands). All reconstructions and measurements are performed with custom-written software using Matlab (The MathWorks, Natick, MA, USA), and results are exported to a data sheet for analysis.

### Primary study parameter

The flexion angle of the TKA in the sagittal plane is measured to determine difference in femoral component placement between the groups. This angle is commonly defined as the angle ‘*γ*’ between the bone stub axis (BSA) of the femur [25] and the neutral line of the femoral component of the prosthesis [12] (Fig. 3). Due to artifacts in the fluoroscopic assessment caused by the metal in the prosthesis, the neutral line is unidentifiable. However, the most anterior plane on the inside of the femoral component of the prosthesis, the frontal flange, can be detected in the fluoroscopic assessment (Fig. 3). Therefore, the angle ‘*θ*’ between the frontal flange and the BSA is measured. The angle between the frontal flange and the neutral line is 97°; thus, the formula *γ* = *θ* − 7° can be used for future comparison between gamma and theta measurements. To obtain the angle ‘*θ*’, the BSA is aligned with the vertical *y*-axis by coronal and sagittal rotating the 3D volume image. Furthermore, the transversal plane is rotated towards an anatomic orientation to correct any femur rotation deviations from acquisition. The latter is necessary to be able to compare different cases. Once the orientation of the femur is standardized within the 3D volume image, the frontal flange can be manually annotated and compared with the *y*-axis, the BSA. For annotation, the sagittal slice with the most prominent frontal flange is chosen and to minimize measurement error from manual annotation an average over 5 iterations is used. We hypothesize that the flexion angle will be larger in the flex rod group (i.e., better fit to the distal anatomy of the femur, Fig. 2).

### Secondary study parameters

The sagittal profile is calculated as the length of lines connecting the intersection of the posterior condylar offset of the distal femur in the sagittal plane (PCOi), and the outer border of either the native femur or femoral component (Fig 4a). The PCOi must be at the exact same location before and after TKA to allow comparison. For this reason, the 3D volume images from before and after TKA are aligned using an automatic intensity-based images registration algorithm in Matlab, based on the shaft of the femur. For constant measurements, the exact same slice has to be selected in every subject. To achieve these constant measurements we pre-align a random 3D volume image with a prosthesis as calibration set. The 3D volume images after TKA are aligned with this calibration set using the same intensity-based image registration algorithm. Subsequently, the pre-operative images (without TKA) are aligned using the same transformation variables.

Due to artifacts in the 3D volume images, the sagittal profile of the trochlea is unidentifiable and sagittal profiles of the condyles are affected. For the best representation of the sagittal profiles, the original designs of the manufacturer are converted into a 3D volume image and added with the pre-aligned calibration set. To avoid image registration errors a pre-aligned calibration set is created for each prosthesis size.

The sagittal profiles of the native femur and the TKA will be calculated at 3 slices: (Fig. 4b) (1) the slice through the most distal point on the medial condyle, oriented at 17.1% of the prosthesis width from the medial side; (2) the slice through the trochlea at the most cranial point of the distal femur, oriented at exactly the middle of the prosthesis; and (3) the slice through the most distal point on the lateral condyle, oriented at 17.1% of the prosthesis width from the lateral side.

One goal of the TKA procedure is restoration of the joint line. This would mean that the amount of implant material at the medial and lateral condyle in full extension of the knee is ± 1 mm from the native situation (DIST, Fig. 4b). A second goal is restoration of the flexion radius. This is operationalized as the amount of implant material at straight posterior at 90° of knee flexion (POST, Fig. 4b) which should be the same as in the native situation (± 1 mm). For mid flexion stability (at 30° of knee flexion), it is also important that the amount of implant material equals the native situation (FLEX30, Fig. 4b). Patellofemoral function can be determined at the trochlear slice. At PCO straight anterior (ANT, Fig. 4b), the amount of implant should equal the amount of native bone (± 1 mm). When the knee flexes, the patella rests on the medial and lateral condyles instead of in the trochlea. So also on the medial and lateral slices at 30° from straight anterior, the length will be calculated (ANT30, Fig 4b). The lengths of the sagittal profile lines are automatically calculated based on pixel density as representative for bone density. In case the segmentation of the femur is incorrect due to artifacts (caused by other bone density than the algorithm expects due to osteoporosis, sclerosis, lower radiation dose, etc.), the lengths are manually corrected. For all five lines, the length in the native situation is subtracted from the length in the component, resulting in a positive sign when the implant is bigger than the native femur (i.e., “overstuffing”), and a negative sign when the implant is smaller than the native femur (i.e., “understuffing”). The margins are defined as ± 1 mm (range of 2 mm). We hypothesize that the differences of the anterior and posterior sagittal contours on the three slices between the pre-operative situation and the post-operative image will be smaller in the flex rod group (i.e., better fit). In the images of the native femur, articular cartilage cannot be seen [20]. Therefore, an average cartilage thickness of 2 mm will be added to the lengths in the native femur [20].

### Functional outcome

Functional outcome will be measured using (1) the timed Get-up and Go test, to measure functional mobility [27]; (2) the stair climbing test to measure ability to ascend and descend a flight of stairs of 14 steps [28]; (3) the Leg Extensor Power Rig (Queens Medical Centre, Nottingham, UK) to measure knee power output [4, 15]. The rig consists of a seat and footplate connected via a lever and chain to a flywheel. Application of force accelerates the flywheel from rest and output is recorded as both maximal wattage (W) generated and as relative power-bodyweight ratio (%) of a single leg extension. The power output is recorded five times per leg and the highest output of the leg in study will be used for analysis and (4) Oxford Knee Score [10], KOOS-PS [26], KUJALA patella score [22], and Knee Society Score [19] to measure patient and clinician reported outcomes. Table 2 shows the schedule for the functional outcome measurements. The hypothesis is that patients in the flex rod group will have better results on all functional outcome scores.

**Table 2:** The surgical study parameters

Parameter	Scale or units	Scored by	Details
Sagittal thickness of the bone cuts during surgery	mm	Surgeon	The bone cuts are posterior cut of patella, anterior cut medial condyle, anterior cut trochlea, anterior cut lateral condyle, anterior 45° cut medial condyle, anterior 45° cut trochlea, anterior 45° cut lateral condyle, distal cut of medial condyle, distal cut of lateral condyle, posterior cut of medial condyle, and posterior cut of lateral condyle. Measured using a Vernier caliper.
Cartilage score of the bone cuts	No, partial, good	Surgeon	See above for the bone cuts
Area of bone cuts	mm^2^	Researcher	All bone cuts are placed with the cut side facing a standardized reference sheet, photographed, and post-operative the area is measured using ImageJ
Cut bone not covered by implant, in mediolateral direction by medial and lateral coverage with implant	mm	Surgeon	Measured per-operative by the surgeon using a Venier caliper. This distance will be measured at the intersection of the anterior chamfer cut and the neutral of the component, at the ventral part of the indention on the component and at the intersection of the neutral of the component and the dorsal chamfer cut
TKA procedure time	min	n/a	Collected post-operatively from hospital information system
Recut or sizing problem	n/a	Surgeon	
Number of releases of collateral ligaments	n/a	Surgeon	
Size of components	n/a	n/a	Collected post-operatively from hospital information system
Intra-operative complications	n/a	Surgeon	
Tourniquet time	min	n/a	Collected post-operatively from hospital information system

### Other study parameters

#### Baseline information

The following demographic and disease-related data will be collected pre-operatively: age, gender, height, weight, strength (Medical Research Council scale), physical activities (UCLA activity score [2]), primary diagnosis, side, alignment of involved knee, and previous surgery of the affected knee.

#### Operative information

See Table 2 for an overview of the parameters recorded during surgery.

#### Radiological information

The following radiological parameters will be measured (see Table 3 for an overview of the radiology schedule): (1) posterior condylar offset (PCO) measured on regular true lateral X-ray [5], (2) patellar tendon angle (PTA) measured on weight bearing with 45° flexion lateral X-ray; (3) lateral patellar tilt [23], and lateral patellar displacement of patellofemoral joint measured on supine sky-line patella X-ray [14], (4) Slope of the tibial component with respect to the tibia measured on the post-operative lateral X-ray.

**Table 3:** Schedule of functional outcome and radiological measurements

Parameter	Visit			
	Pre-operative	3 months	12 months	24 months
Fit (flexion angle and sagittal profile)	Multi Diagnost Eleva, supine	Multi Diagnost Eleva, supine	-	-
Functional outcome	Time Get-up and Go test, stair climbing test, Leg Extensor Power Rig	Time Get-up and Go test, stair climbing test, Leg Extensor Power Rig	Time Get-up and Go test, stair climbing test, Leg Extensor Power Rig	Time Get-up and Go test, stair climbing test, Leg Extensor Power Rig
Posterior condylar offset	Lateral x-ray, standing	Lateral x-ray, standing	Lateral x-ray, standing	-
Patellar tendon angle	Lateral x-ray, standing, 45° flexion	Lateral x-ray, standing, 45° flexion	Lateral x-ray, standing, 45° flexion	-
Patellar tilt and displacement	Sky-line patella x-ray, supine	Sky-line patella x-ray, supine	Sky-line patella x-ray, supine	-

All patients will receive post-operative standard care according to the usual practice of our hospital. Post-operative blood loss and/or complications will be documented on the case report form. Post-operative data through time of hospital discharge will be reported on the discharge form.

#### Statistical analysis

Sagittal flexion angle of the TKA will be reported as mean and standard deviation (or median and interquartile range (IQR)). An independent two-sample *t* test (or non-parametric equivalent) will be used to analyze the effect of group (flex rod versus standard rod). All secondary outcome parameters will be reported as mean and SD (or median and IQR). Repeated mixed model analysis techniques will be used to analyze the effects of group, time, and their interaction. All other study parameters will be reported as mean and standard deviation (or median and IQR). Independent *t* tests (or non-parametric equivalent) and Chi square (in case of frequencies) will be used to analyze the effect of group. All statistical analyses will be performed using Stata/IC for Windows, Release 13 (StataCorp LLC, College Station, TX, USA).

The sample size calculation is based on the primary study parameter: flexion angle of the prosthesis in the sagittal plane. Our hypothesis is that this angle at 3 months will be smaller in the standard rod group compared to the flex rod group. First, the flexion angle in posterior stabilized TKA has been reported to be about 6° [1]. Second, the mean intra-observer difference between measurements for this angle is 2.3° with an estimated standard deviation of 5° [16]. We assume that a clinical relevance of a mean difference of 5° between the rod groups can be measured [21], but this will be further studied in our study. Using G*Power (version 3.1.7 [11]), a sample size was calculated for an independent two-sample *t* test, with the variables *m*1 = 6°, *m*2 = 11°, *σ*1 = 5°, *σ*2 = 5°, *α* = 0.05, and power = 0.80. This calculation yielded a sample size for the total group of *N* = 54, with 27 for each group. Taking into account a drop out of participants during the course of the study of 10%, a sample size of *N* = 60 subjects is needed, with 30 subjects in each group.

## Discussion

This randomized controlled trial has been set up to assess how the use of a flex rod affects the fit of the TKA and if this results in better functional outcome. As the sagittal fit of TKA is not a standardized outcome measure, we chose to define the fit with two outcome measures: the first being the flexion angle of the femoral component. It is expected that the flexion angle will be larger in the flex rod group as the femoral component is expected to have a better fit to the distal anatomy of the femur. The second outcome measure is the contour of the distal femur, quantified in the anterior sagittal profile and the posterior sagittal profile at various positions. It is expected that for the flex rod group, due to smaller sizing of the femoral component, the distal anatomy of the femur with TKA will be closer to the pre-operative distal anatomy. We expect that this will be most apparent in the anterior profile where the effect of overstuffing after use of the standard rod is expected to be largest [17]. Also, for this reason, patients in this trial will receive a posterior stabilized TKA, standardizing the posterior placement of the femoral component (in contrast to a cruciate-retaining TKA), and with differences in the anterior part of the distal femur as the only degree of freedom.

Custom-made software is used for calculating the parameters of sagittal fit of the TKA. Due to the unavailability of existing software for measuring the specific parameters for the specific model of TKA, this software was produced in-house. The advantages of this software is that it is tailored made for the study-specific parameters. Disadvantages of this software are that currently we have no data on the reliability and validity of the measurements and that by using imaging of the knee, it is unable to account for articular cartilage thickness [20]. During the TKA procedure, the cartilage of the different cuts will be scored. Using this information, the measurements of the sagittal profile can be corrected for cartilage thickness of 0, 1, and 2 mm, with the values based on the literature [20]. Other parameters that are measured during the TKA procedure are the sagittal thickness (in millimeters) of the cuts, and the area of the cuts. It is expected that due to a better fit of the femoral component to the distal anatomy in the flexible IM rod group, less bone has to be cut, resulting in lower thickness and smaller area of these cuts.

We expect that the use of the flex rod will result in an overall better functional outcome. This is supported by Hitt et al. [17], who studied patient and clinician reported outcome measures in a RCT. The modeling study by Marra et al. [24] showed that such effects may be explained by the increase in the patellar tendon moment arm. Our aim is to study the effects of a flex rod in a prospective RCT combining imaging, surgery details, functional outcome, and patient-reported outcomes, thus providing a data set on which our hypotheses can be thoroughly tested.

## Data Availability

Not applicable.

## Abbreviations

ADL: Activities of daily living
BSA: Bone stub axis
IQR: Interquartile range
PCO: Posterior condylar offset
PCOi: Intersection of the posterior condylar offset of the distal femur in the sagittal plane
PTA: Patellar tendon angle
RCT: Randomized controlled trial
TKA: Total knee arthroplasty
